# Supplementary material for: Phospho-seq: integrated, multi-modal profiling of intracellular protein dynamics in single cells
Source: Nat Commun. 2025 Feb 4;16:1346. doi: 10.1038/s41467-025-56590-7 (PMC11794950; doi:10.1038/s41467-025-56590-7)
Supplement: Supplementary file 1 — Supplementary Information [file 41467_2025_56590_MOESM1_ESM.pdf]

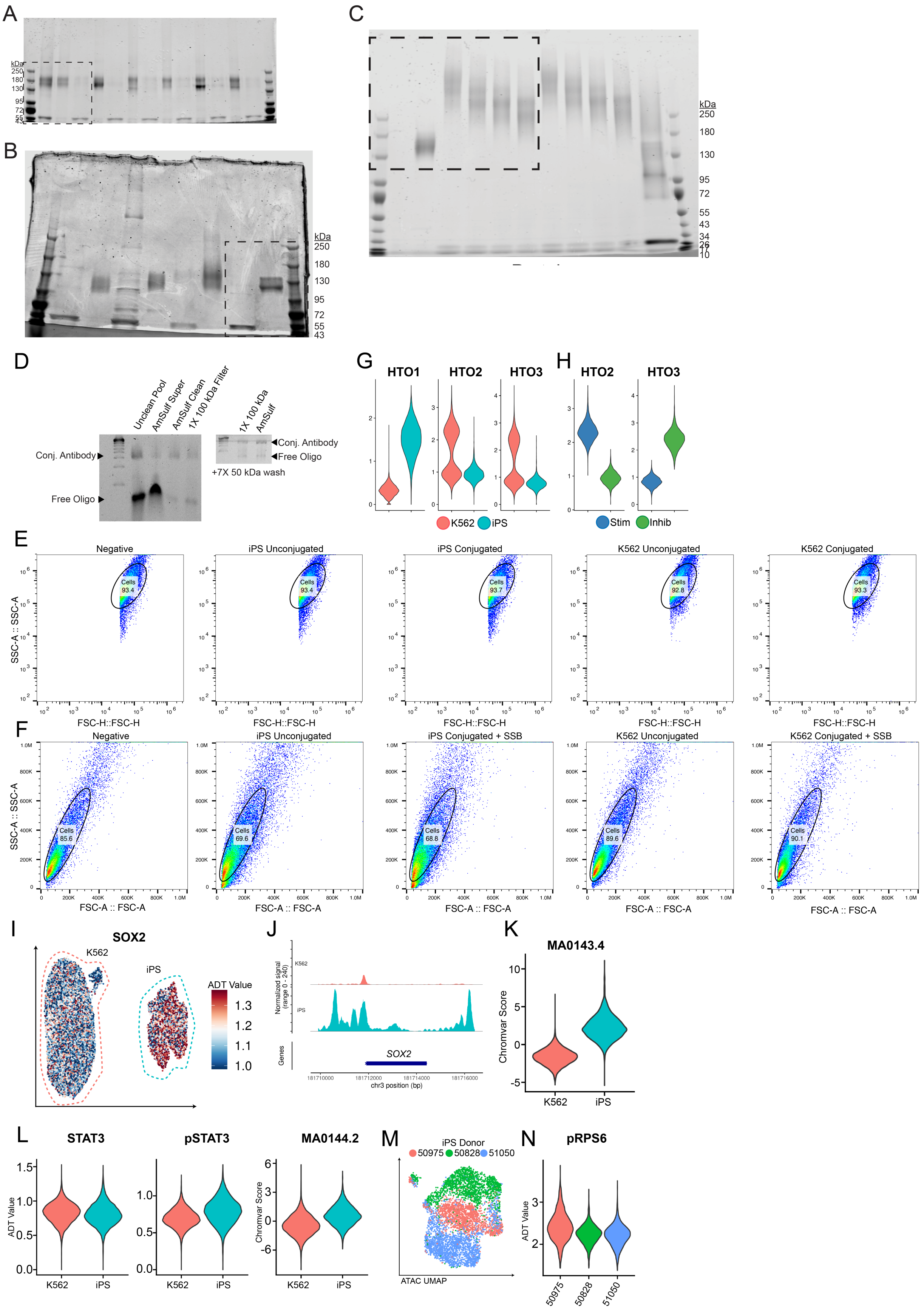

A
kDa
250
180
130
95
72
55
43
B
kDa
250
180
130
95
72
55
43
C
kDa
250
180
130
95
72
55
43
34
26
17
10
D
Unclean Pool
AmSulf Super
AmSulf Clean
1X 100 kDa Filter
1X 100 kDa
AmSulf
Conj. Antibody
Free Oligo
Conj. Antibody
Free Oligo
+7X 50 kDa wash
G
HTO1
HTO2
HTO3
K562
iPS
H
HTO2
HTO3
Stim
Inhib
E
Negative
iPS Unconjugated
iPS Conjugated
K562 Unconjugated
K562 Conjugated
Cells 93.4
Cells 93.4
Cells 93.7
Cells 92.8
Cells 93.3
SSC-A :: SSC-A
FSC-H::FSC-H
F
Negative
iPS Unconjugated
iPS Conjugated + SSB
K562 Unconjugated
K562 Conjugated + SSB
Cells 85.6
Cells 69.6
Cells 68.8
Cells 89.6
Cells 90.1
SSC-A :: SSC-A
FSC-A :: FSC-A
I
SOX2
K562
iPS
ADT Value
1.3
1.2
1.1
1.0
J
Normalized signal (range 0 - 240)
K562
iPS
Genes
SOX2
181710000
181712000
181714000
181716000
chr3 position (bp)
K
MA0143.4
Chromvar Score
K562
iPS
L
STAT3
pSTAT3
MA0144.2
ADT Value
Chromvar Score
K562
iPS
M
iPS Donor
50975
50828
51050
ATAC UMAP
N
pRPS6
ADT Value
50975
50828
51050

Supplementary Fig. 1: Supplementary Information for Figure 1. a) Uncropped gel scan from Figure 1C. Dotted lines indicate area of cropping. **b)** Uncropped gel scan from Figure 1C. Dotted lines indicate area of cropping. **c)** Uncropped gel scan from Figure 1D. Dotted lines indicate area of cropping. **d)** DNA electrophoresis gel of antibody pool at different indicated stages of clean-up showing the degree of remaining free oligo after filtration steps. **e**) Density plots showing the gating for cells sorted in Fig 1E, left panel **f)** Density plots showing the gating for cells sorted in Fig 1E, right panel **g)** Violin plot of HTO expression for each HTO in K562 and iPS cells. **h)** Violin plot of HTO expression for each HTO in K562 cells classified from stimulated or inhibited conditions. **i)** UMAP representation of K562 and iPS cells colored by normalized ADT values for SOX2. **j)** Coverage plot of chromatin accessibility of K562 and iPS cells at the SOX2 genomic locus. **k)** Violin plot of chromVAR scores for the SOX2 binding motif (MA0143.4) in K562 and iPS cells. **l)** Violin plots of normalized ADT values for STAT3 (left panel) pSTAT3 (middle panel) and STAT3 motif chromVAR scores (right panel) for K562 and iPS cells. **m)** UMAP representation of iPS cells colored by iPSC donor based on scATAC-sequencing data. **n)** Violin plot of normalized ADT values of pRPS6 split by iPSC donor identity.

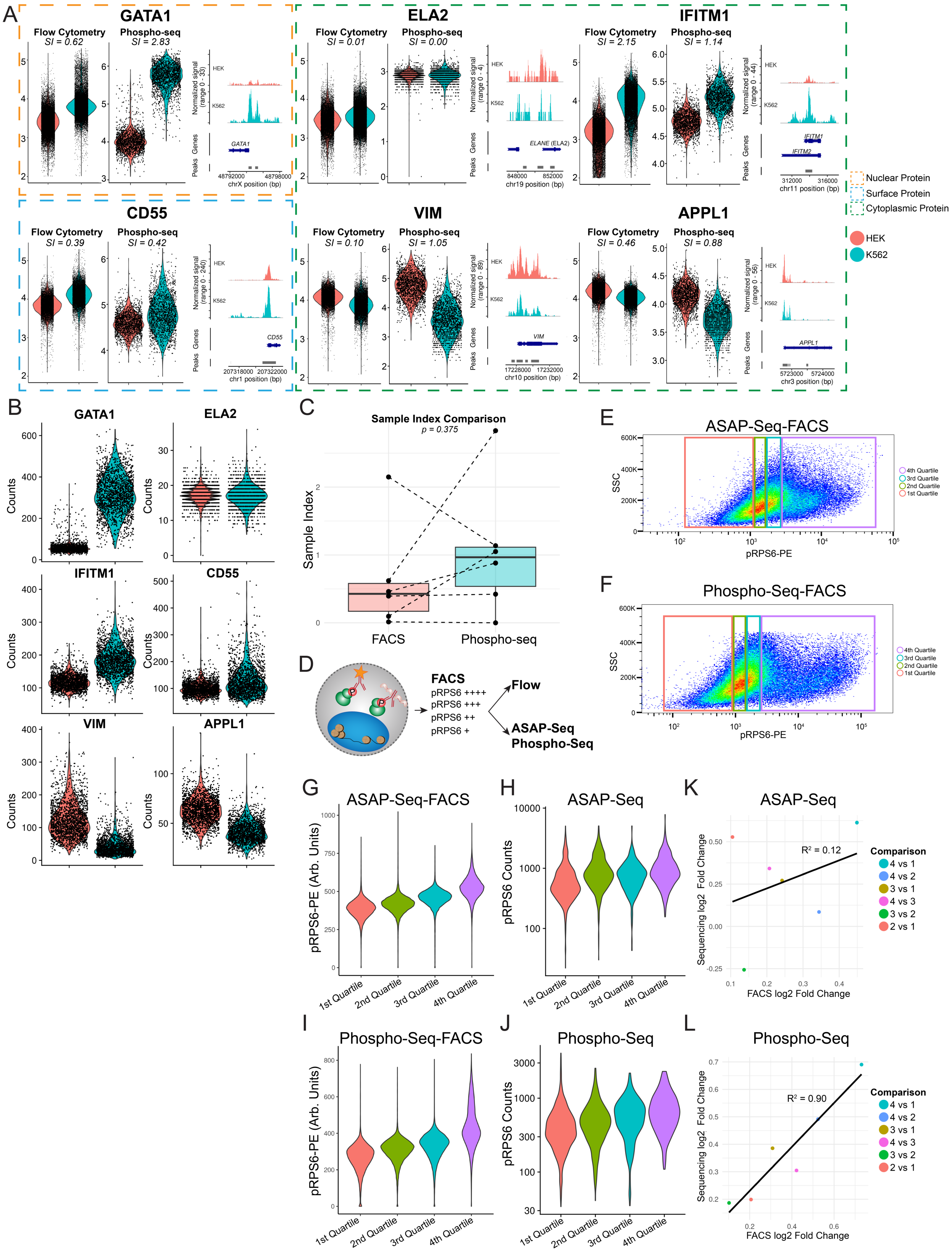

Supplementary Fig. 2: Benchmarking Phospho-seq against comparable technologies. a) Violin plots and corresponding chromatin accessibility plots (right) from indicated intracellular protein quantification method, Flow

Cytometry (left) or Phospho-seq (middle), across six proteins. Flow cytometry measurements are quantified on a Log Scale. SI refers to the “staining index” (methods) **b)** Violin Plots showing the raw counts for each antibody in each cell type from the Phospho-seq experiments in (a) **c)** Paired boxplot comparing the staining indices from each experiment in (a). P-value is calculated through a paired t-test. Boxes represent 25th-75th percentile and the line represents the median. Whiskers indicate 1.5X the upper and lower Interquantile Range. While the increased SI of Phospho-seq is not statistically significant, this shows that data quality from Phospho-seq matches data quality from gold-standard approaches (Flow Cytometry). **d)** Schematic of experimental paradigm for benchmarking Phospho-seq against ASAP-seq **e)** Density plots of FACS sorted cells and bins collected for ASAP-seq-FACS. **f)** Density plots of FACS sorted cells and bins collected for Phospho-seq-FACS. **g)** Violin plots of pRPS6 levels from each bin from the ASAP-seq experiment as measured by Flow Cytometry. **h)** Violin plots of pRPS6 levels from each bin from the ASAP-seq experiment as measured by ASAP-seq. **i)** Violin plots of pRPS6 levels from each bin from the Phospho-seq experiment as measured by Flow Cytometry. **j)** Violin plots of pRPS6 levels from each bin from the Phospho-seq experiment as measured by Phospho-seq **k)** Scatter plot comparing log2 fold changes between bins from flow cytometry compared to ASAP-seq. **l)** Scatter plot comparing log2 fold changes between bins from flow cytometry compared to Phospho-seq.

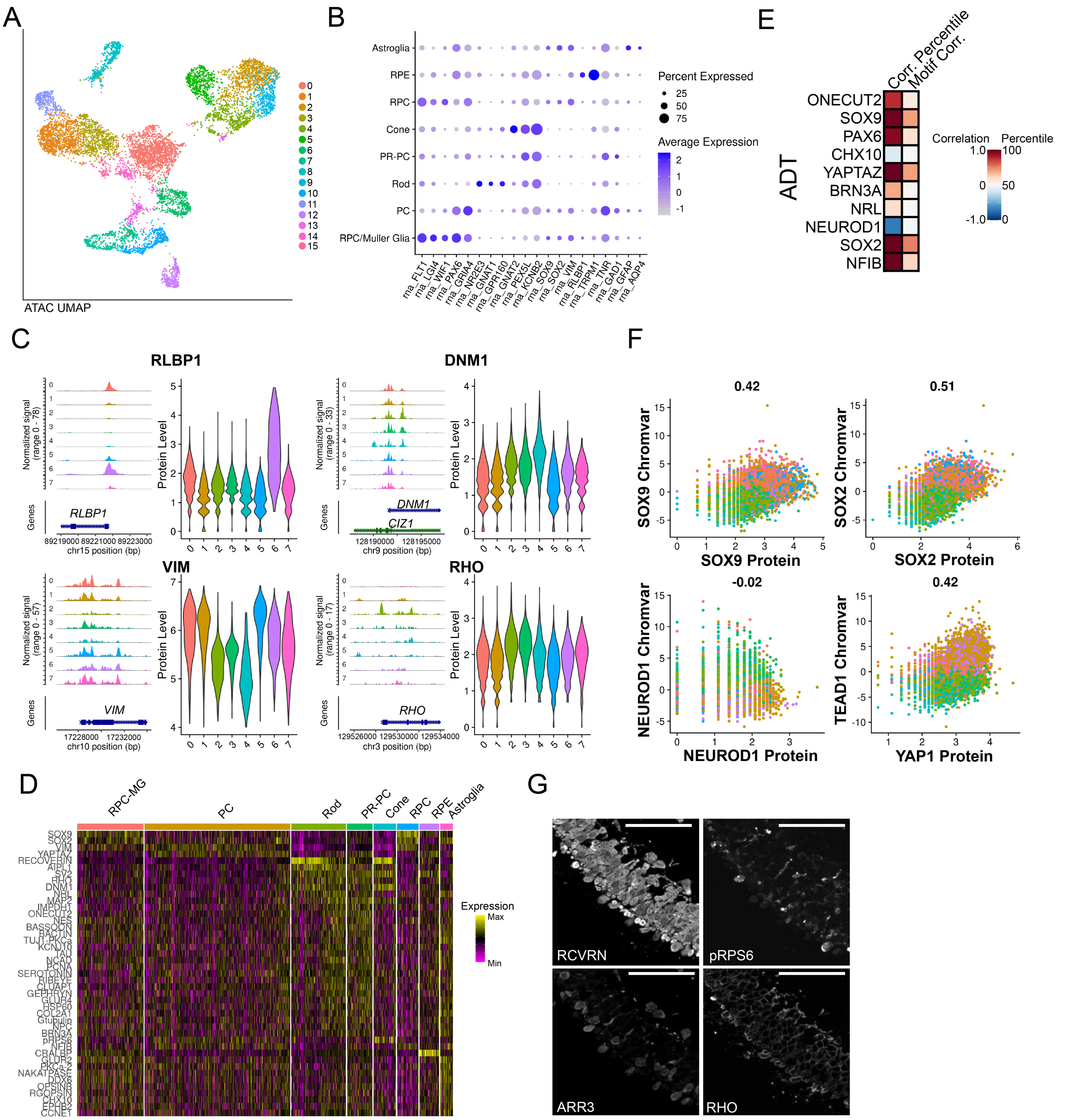

Supplementary Fig. 3: Supplementary Information for Figure 2. a) UMAP representation of cells colored by unsupervised clustering based on scATAC-Seq modality in Phospho-seq of retinal organoids. **b)** Dotplot showing the gene activity scores of marker genes and the percentage of cells they are expressed in from each assigned cell type identity in retinal organoids. **c)** Coverage and violin plots of the gene promoter and protein level respectively of RLBP1,DNM1,VIM and RHO. Color and order are the same as in **Fig2B**. **d)** Heatmap showing scaled ADT expression for all differentially expressed (minimum log2 fold change >0.25) ADTs organized by assigned cell type. **e)** Heatmap showing absolute correlation and correlation percentile between ADT expression and TF chromVAR score for each ADT-associated TF across all cells. **f)** Scatter plots showing the correlation between chromVAR scores and protein measurements for select transcription factors. **g)** Confocal images of 33 week old retinal organoids harvested and immunostained with antibodies against the photoreceptor marker RCVRN, cone marker ARR3, rod marker RHO and pRPS6. Scale bars are 50 μm.

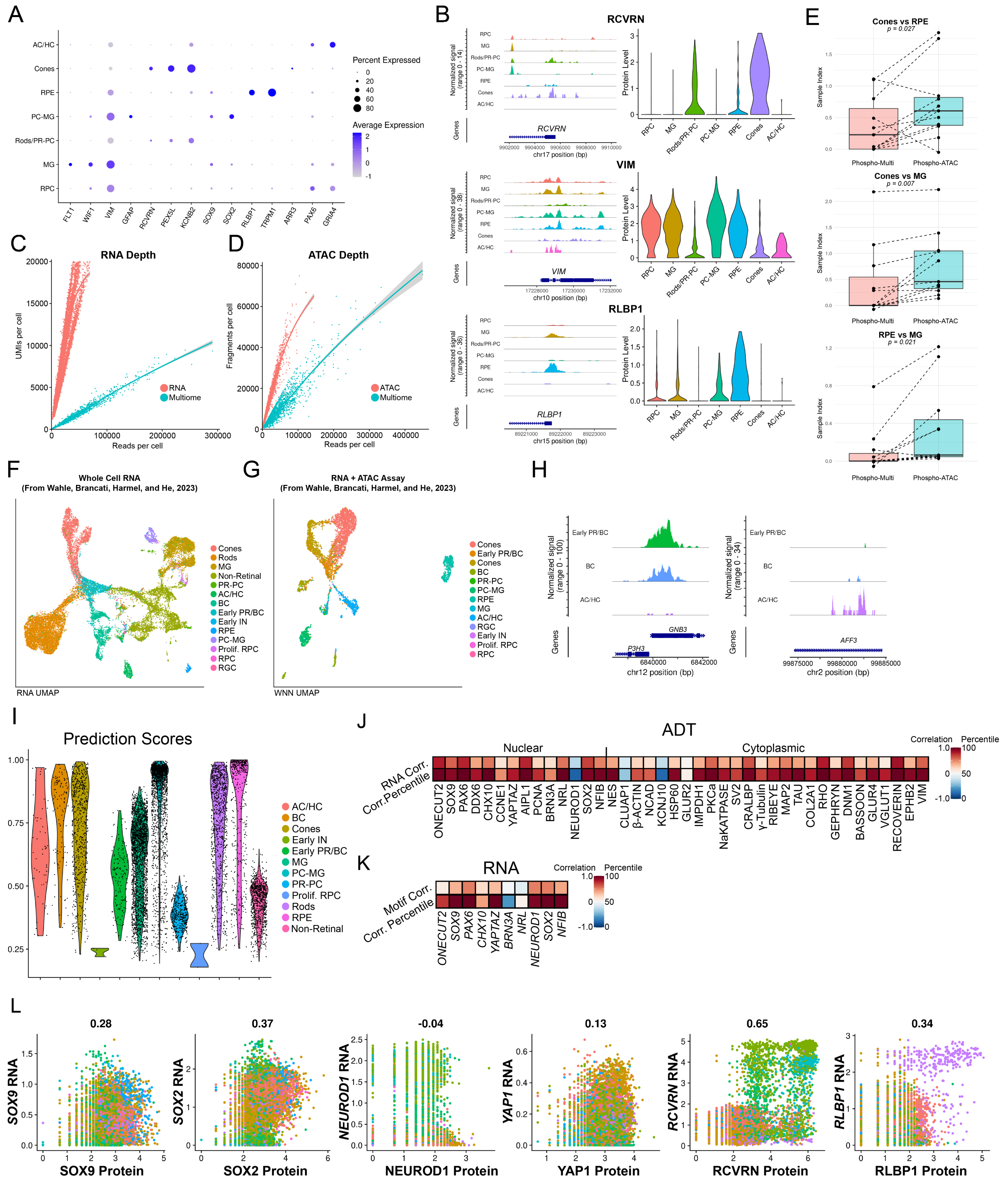

Supplementary Fig. 4: Supplementary Information for Figure 3. a) Dotplot showing the gene expression of marker genes and the percentage of cells they are expressed in from each cell identity from Phospho-seq-multi experiment. **b)** Coverage and violin plots of the gene promoter and protein level respectively of RCVRN, VIM and RLBP1 from the Phospho-seq-multi experiments. **c)** Scatter plot comparing number of RNA UMIs captured vs. number of RNA reads in Phospho-seq with the 10X 3'RNA-kit vs. 10X Multiome. **d)** Scatter plot comparing number of ATAC fragments captured vs. number of ATAC reads in Phospho-seq with the 10X scATAC-kit vs. 10X Multiome. **e)** Paired boxplots comparing the staining indices of ADTs between Phospho-seq-multi and Phospho-seq experiments for Cones vs RPE, Cones vs MG and RPE vs MG. P-value is calculated through a paired t-test. Boxes represent 25th-75th percentile and the line represents the median. Whiskers indicate 1.5X the upper and lower Interquantile Range.

f) UMAP representation of week 38 retinal organoid cells colored by cell type from scRNA-Seq on whole unfixed cells. Data taken from Wahle, Brancati, Harmel and He (2023). **g)** Weighted Nearest Neighbors UMAP representation of week 38 retinal organoid cells colored by cell type from a 10X multiome experiment on unfixed nuclei. Data taken from Wahle, Brancati, Harmel and He (2023). **h)** Coverage plots for GNB3 (left) and AFF3 (right) with transferred cell labels. **i)** Violin Plot of prediction scores per assigned cell type from bridge integration of whole cell RNA dataset with Phospho-seq dataset. **j)** Heatmap showing absolute correlation and correlation percentile between ADT expression and RNA expression for each RNA-associated protein across all cells. **k)** Heatmap showing absolute correlation and correlation percentile between RNA expression and TF chromVAR score for each RNA-associated TF across all cells. **l)** Scatter plots showing correlations of select imputed RNA measurements against their corresponding proteins.

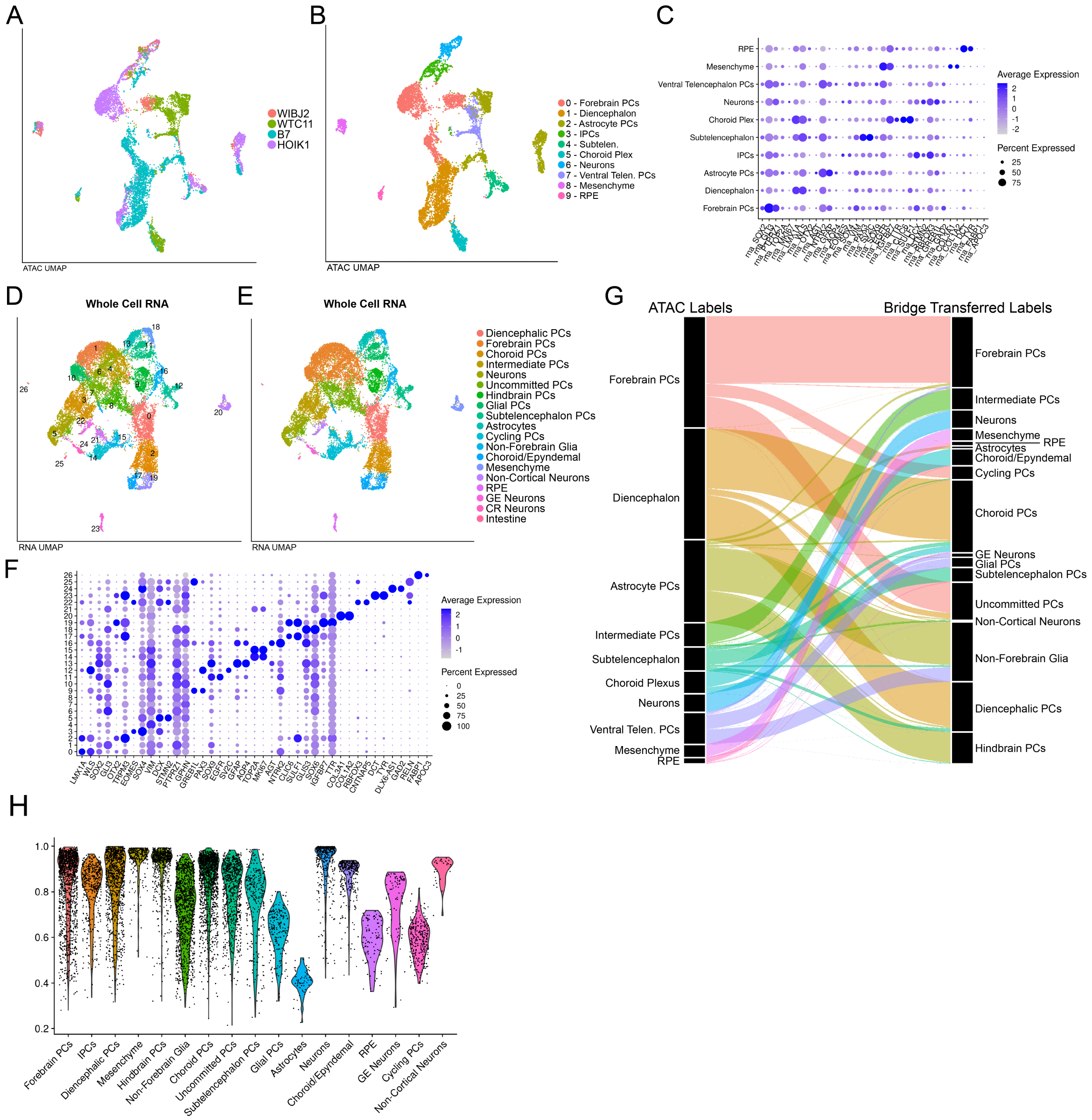

Supplementary Fig. 5: Supplementary Information for Figure 4. a) UMAP representation of brain organoid cells colored by iPSC donor based on scATAC-Seq modality in Phospho-seq. **b)** UMAP representation of cells colored by unsupervised clustering based on scATAC-Seq modality in Phospho-seq. **c)** Dotplot showing the gene activity scores of marker genes and the percentage of cells they are expressed in from each assigned cell type identity in the Phospho-seq dataset. **d)** UMAP representation of cells colored by unsupervised cluster assignment from scRNA-Seq on whole unfixed cells. **e)** UMAP representation of cells colored by cell type assignment from scRNA-Seq on whole unfixed cells. **f)** Dotplot showing the gene expression of marker genes and the percentage of cells they are expressed in from each cluster identity in whole, unfixed cells. **g)** Alluvial plot demonstrating cell label transfer when using bridge integration. **h)** Violin Plot of prediction scores per assigned cell type from bridge integration of whole cell RNA dataset with Phospho-seq dataset.

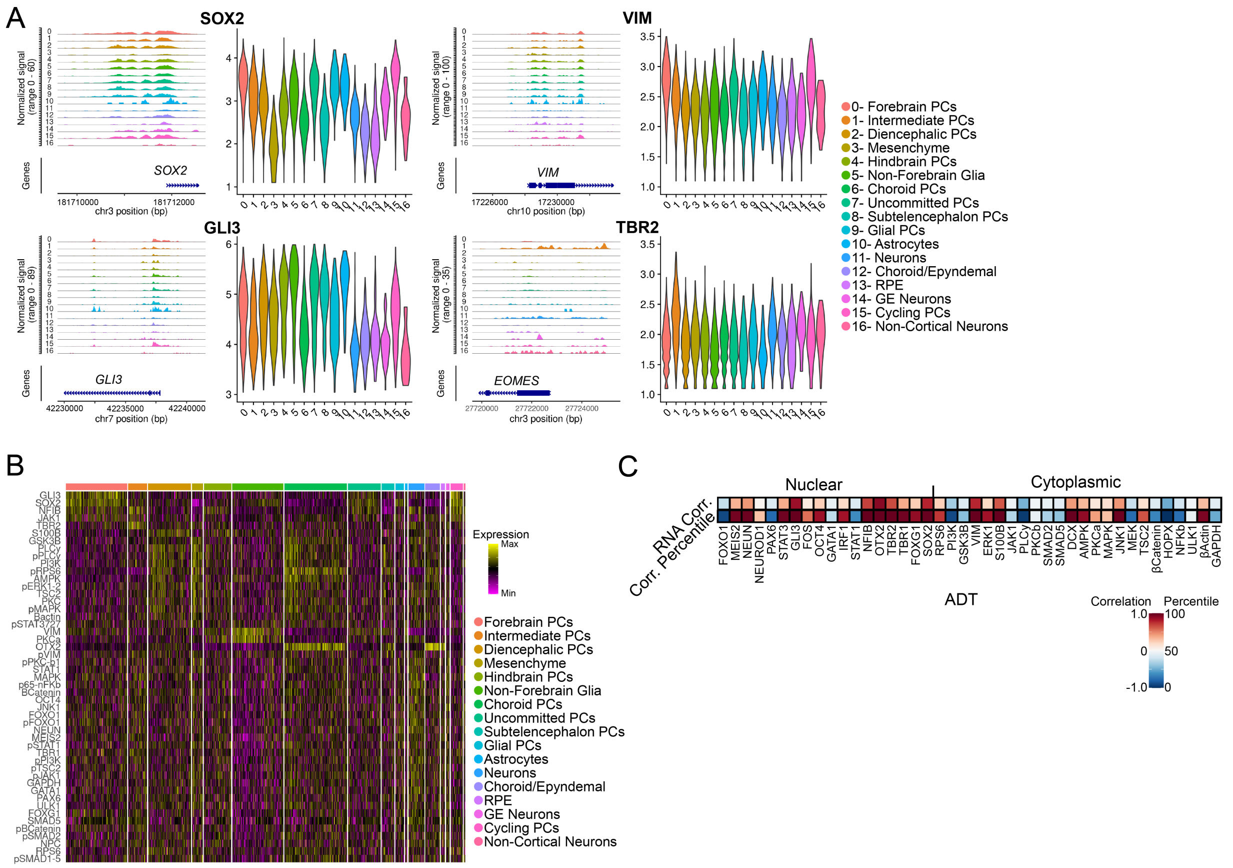

Supplementary Fig. 6: Protein-level data for Brain Organoid Phospho-seq. a) Coverage and violin plots of the gene promoter and protein level respectively of SOX2, VIM, GLI3 and TBR2. Color and order are the same as in **Fig 4D**. **b)** Heatmap showing scaled ADT expression for all differentially expressed (minimum log2 fold change >0.25) ADTs organized by assigned cell type. **c)** Heatmap showing absolute correlation and correlation percentile between ADT expression and RNA expression for each RNA-associated ADT.

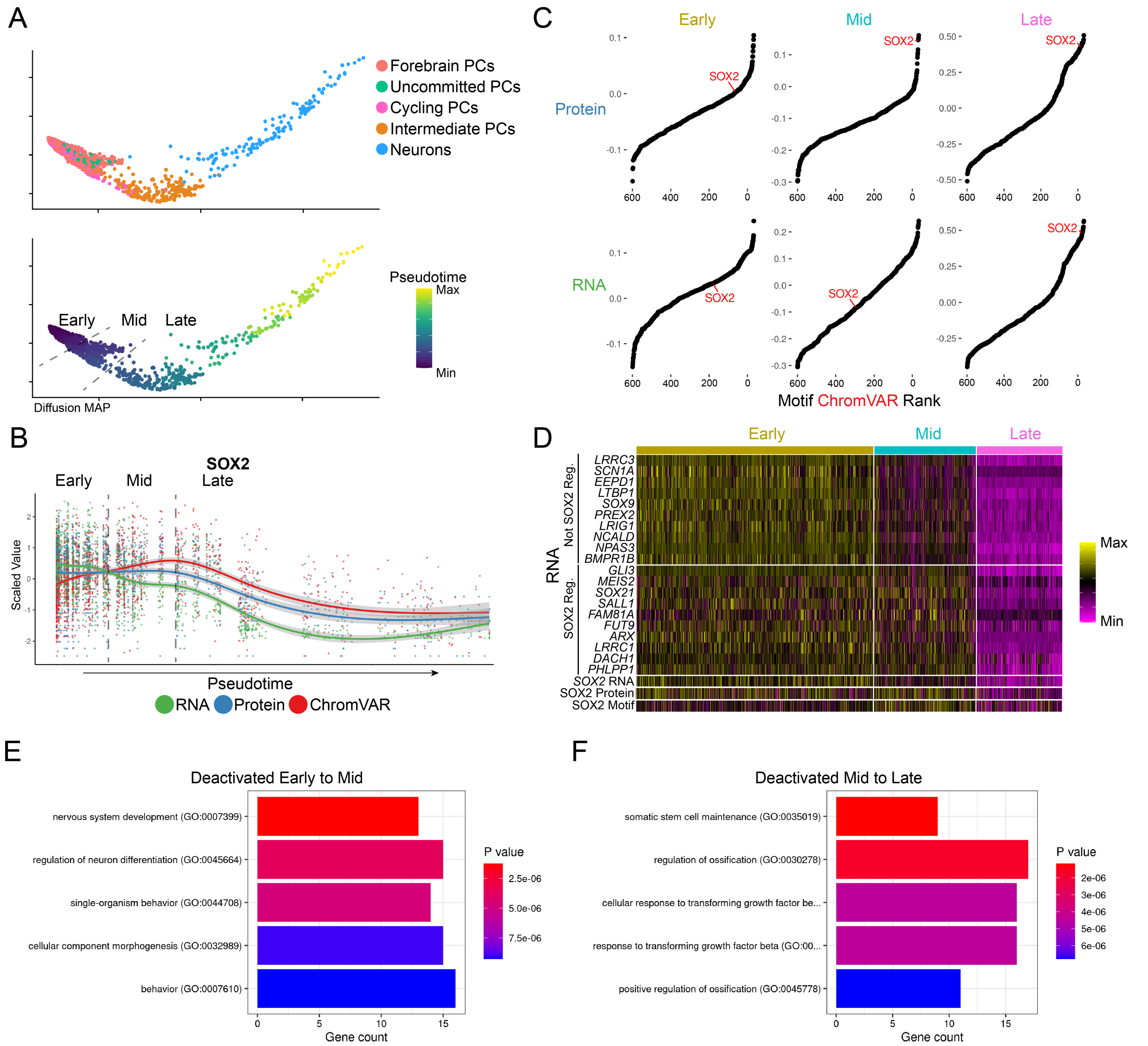

Supplementary Fig. 7: Dynamics of SOX2 in early development. a) Diffusion map of cells differentiating from Forebrain PCs to Neurons colored by cell type (top panel) and pseudotime as determined by monocle (bottom panel). Dashed lines indicate pseudotime cut-offs determined by SOX2 expression and activity. **b)** Scatter plot showing scaled values of SOX2 RNA, protein and motif chromVAR score across pseudotime as determined in (a). Dashed lines indicate the same SOX2-based pseudotime cut-offs as in (a). **c)** Rank-correlation plots of transcription factor motif accessibility vs. SOX2 Protein (top panel) and SOX2 RNA (bottom panel) for each of the pseudotime cut-off group. **d)** Heatmap of a subset of genes not regulated by SOX2 and regulated by SOX2 across pseudotime bins**. e)** Bar plot of the five most significant gene ontology categories associated with the top 500 genes decreasing from early to mid pseudotime bins across forebrain neuronal development. **f)** Bar plot of the five most significant gene ontology categories associated with the top 500 genes decreasing from mid to late pseudotime bins across forebrain neuronal development.

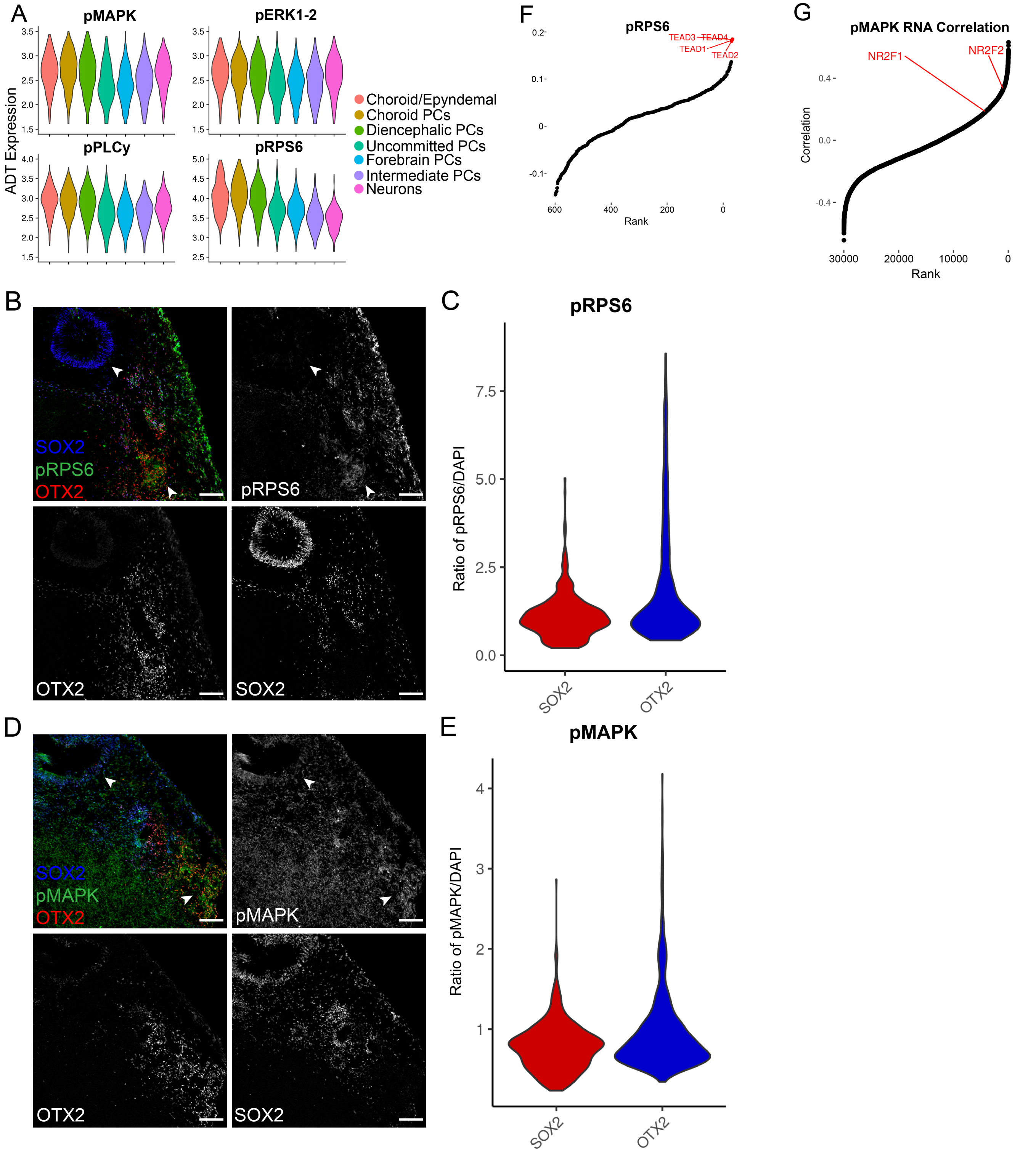

Supplementary Fig. 8: Signaling in Brain Organoids. a) Violin Plots of pMAPK, pERK1-2, pPLCy and pRPS6 across cell types in the diencephalic and telencephalic differentiation trajectories. **b)** Confocal image of B7 iPs cell derived brain organoids stained with OTX2, SOX2 and pRPS6. Scale bar is 200 µm. **c)** Quantification of pRPS6 in 50 µm hexes from image in panel (b) from 415 SOX2+ hexes and 597 OTX2+ hexes. **d)** Confocal image of B7 iPs cell derived brain organoids stained with OTX2, SOX2 and pMAPK. Scale bar is 200 µm. **e)** Quantification of pMAPK in 50 µm hexes from image in panel (d) from 815 SOX2+ hexes and 639 OTX2+ hexes. This experiment was performed once **f)** Rank-correlation plot showing correlation between pRPS6 levels and motif accessibility across the retinalPhospho-seq dataset. The top hits are indicated in red. **g)** Rank-correlation plot of pMAPK ADT signal vs. RNA expression with NR2F1 and NR2F2 highlighted.

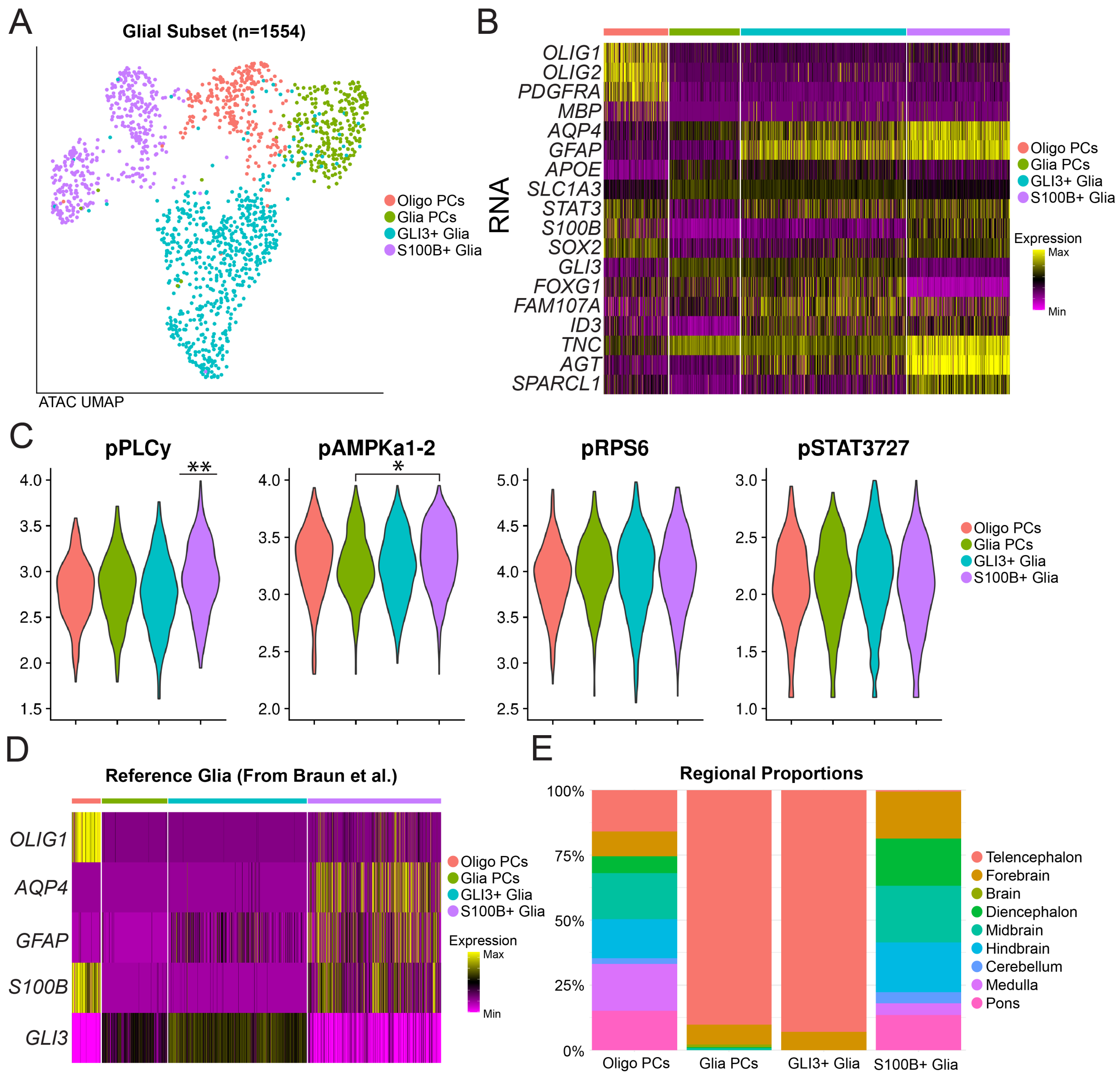

Supplementary Fig. 9: Glial Heterogeneity in Brain Organoids. a) UMAP of reclustered subset of glial cells from 3-month old brain organoid Phospho-seq experiment. **b)** Heatmap of glial marker genes across glial dataset. **c)** Violin plots of ADT levels of pPLCy, pAMPKa1-2, pRPS6 and pSTAT3-727 across glial dataset. * = adjusted p-value of <0.05, ** = adjusted p-value <0.01 using Wilcoxon Rank Sum test. **d)** Heatmap of cell type specific markers of reference mapped glial cells from a first trimester developing brain dataset. **e)** Stacked proportional bar plot of regional identity of reference mapped glial cells.

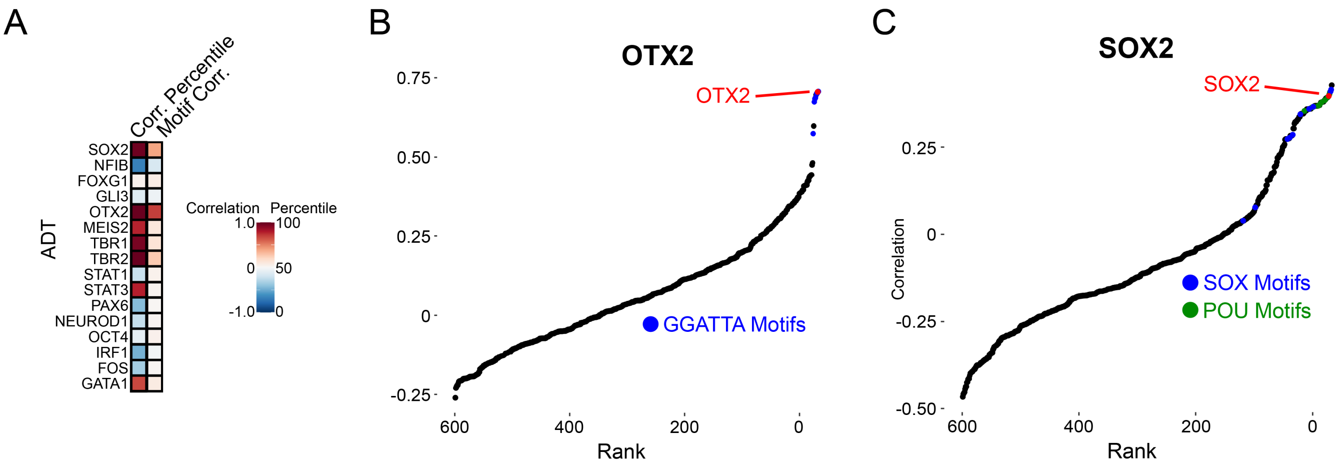

Supplementary Fig. 10: ADT-Motif Correlation in Brain Organoids. a) Heatmap showing absolute correlation and correlation percentile between ADT expression and TF chromVAR score for each ADT-associated TF. **b)** Rank-correlation plots for OTX2 ADT with all transcription factor motifs with OTX2 indicated in red and other similar motifs indicated in blue. **c)** Rank-correlation plots for SOX2 ADT with all transcription factor motifs with SOX2 indicated in red and other similar motifs indicated in blue and green.

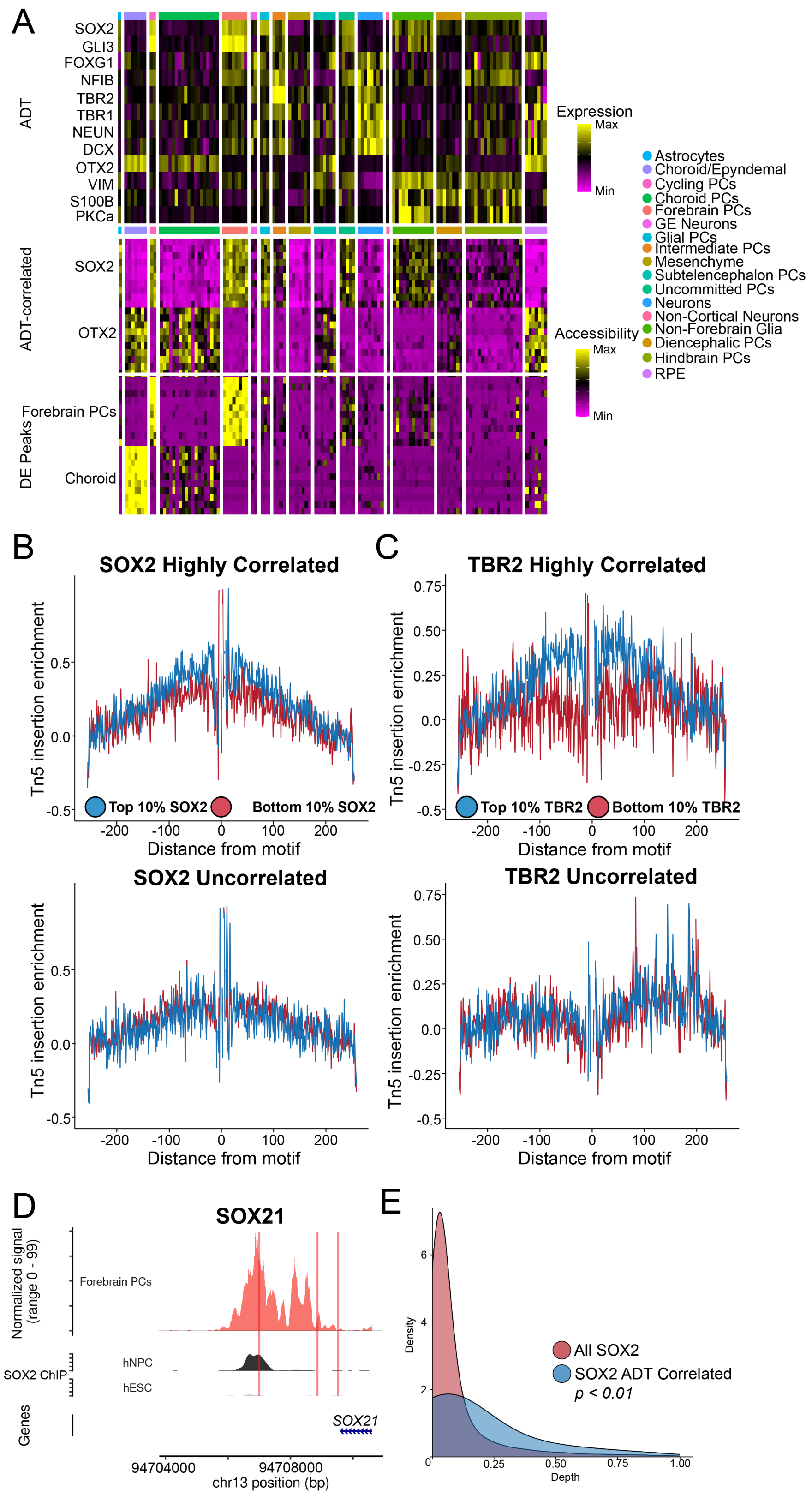

Supplementary Fig. 11: Supporting Information for Figure 6. a) Heatmaps of metacelled data showing normalized marker ADT values (top panel), peaks correlated with SOX2 and OTX2 ADT expression (middle panel) and differentially accessible peaks for Forebrain and Choroid PCs determined from the non-metacelled data. **b)** Tn5 cut-site footprinting between cells with high SOX2 expression and low SOX2 expression in peaks that are highly correlated with SOX2 (top panel) and uncorrelated with SOX2 (bottom panel). **c)** Tn5 cut-site footprinting between cells with high TBR2 expression and low TBR2 expression in peaks that are highly correlated with TBR2 (top panel) and uncorrelated with TBR2 (bottom panel). **d)** Coverage plots of Forebrain PCs from this dataset compared to SOX2 ChIP-seq peaks for human *in vitro* differentiated neural precursors (hNPC) and human hESCs (hESC). **e)** Density plot showing ChIP-Seq depth at all peaks with a SOX2 motif compared to SOX2 ADT Correlated peaks identified in this study. Significance determined by a Wilcoxian rank-sum test

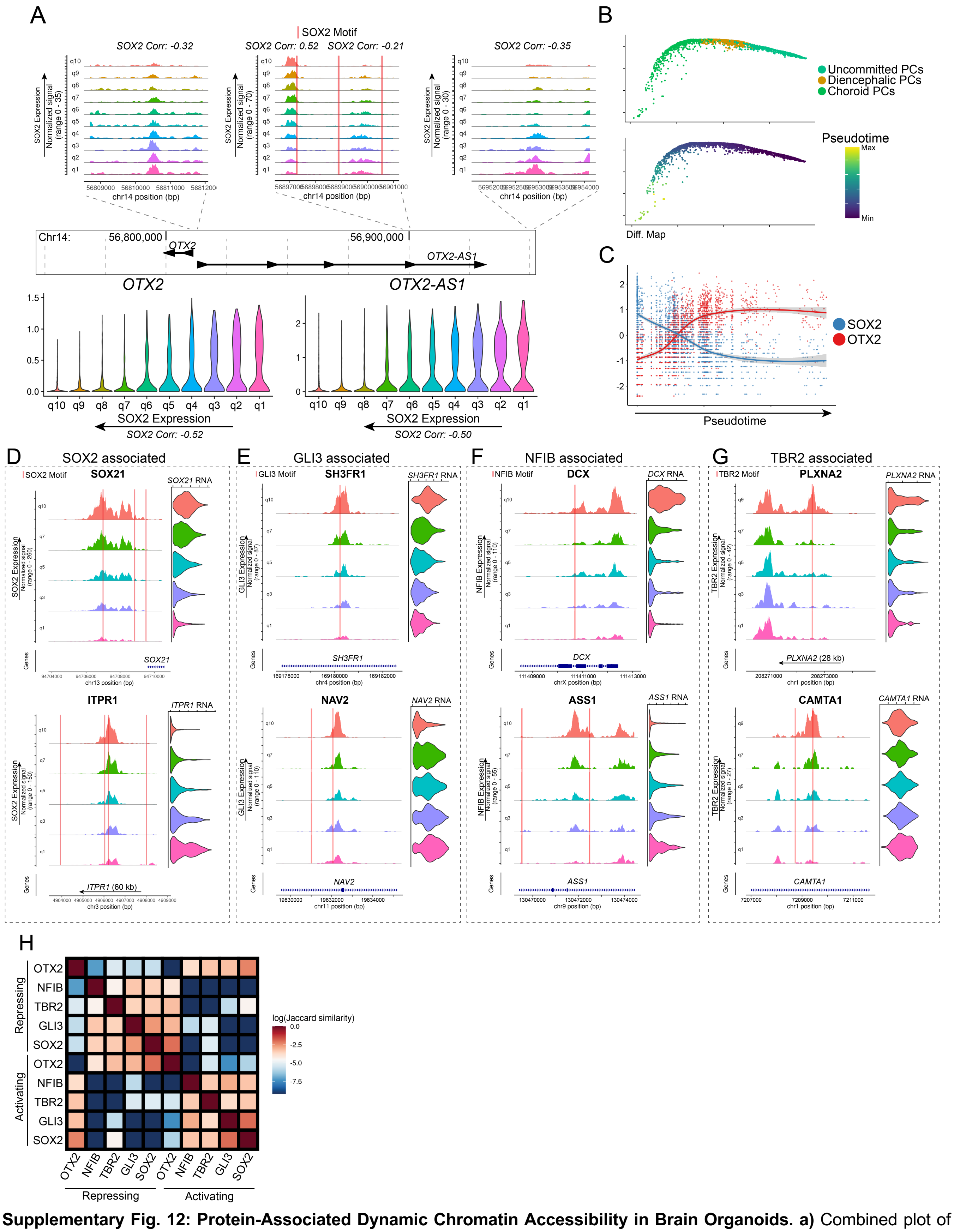

Supplementary Fig. 12: Protein-Associated Dynamic Chromatin Accessibility in Brain Organoids. a) Combined plot of accessibility in peaks proximal to the OTX2 locus and associated gene expression. Plots are organized by quantile ADT expression of SOX2 throughout the whole dataset. Red lines on coverage plots are indicative of SOX2 binding motifs. **b)** Diffusion map of cells differentiating from Uncommitted PCs to Choroid PCs colored by cell type (top panel) and pseudotime as determined

by monocle (bottom panel). **c**) Scatter plot showing scaled values of SOX2 and OTX2 protein across pseudotime as determined in (b). **d)** Example of an inferred transcription activating (top) and repressing (bottom) peak associated with SOX2. Coverage plots and violin plots are ordered by quantile ADT expression for SOX2. Red lines indicate the location of a SOX2 binding motif. **e)** Same as in (a) but for GLI3. **f)** same as in (a) but for NFIB. **g)** same as in (a) but for TBR2. **h)** Jaccard similarity matrix of inferred activating and repressing peaks from each of the TFs highlighted.

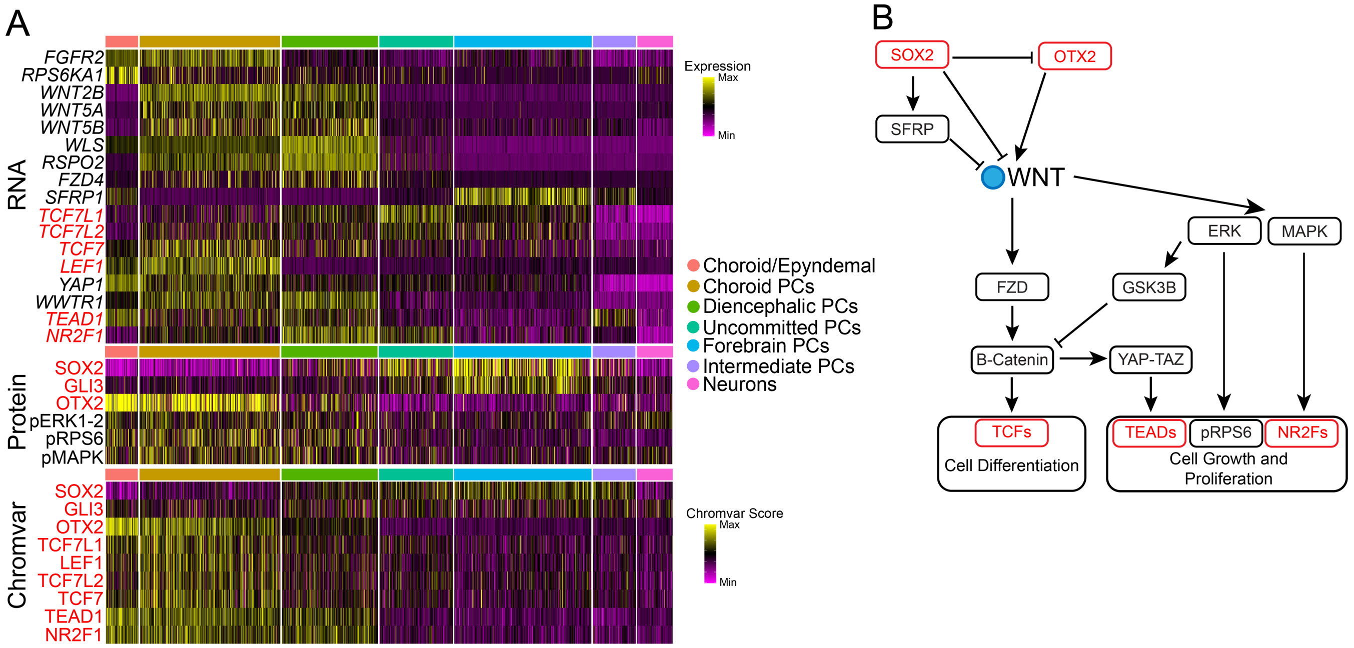

Supplementary Fig. 13: WNT signaling in Brain Organoids. a) Heatmap of WNT-signaling related gene expression (top panel), protein expression (middle panel) and chromVAR scores for WNT-signaling related transcription factors. **b)** Schematic of regulation of WNT signaling during diencephalic and telencephalic differentiation based on Phospho-seq data.

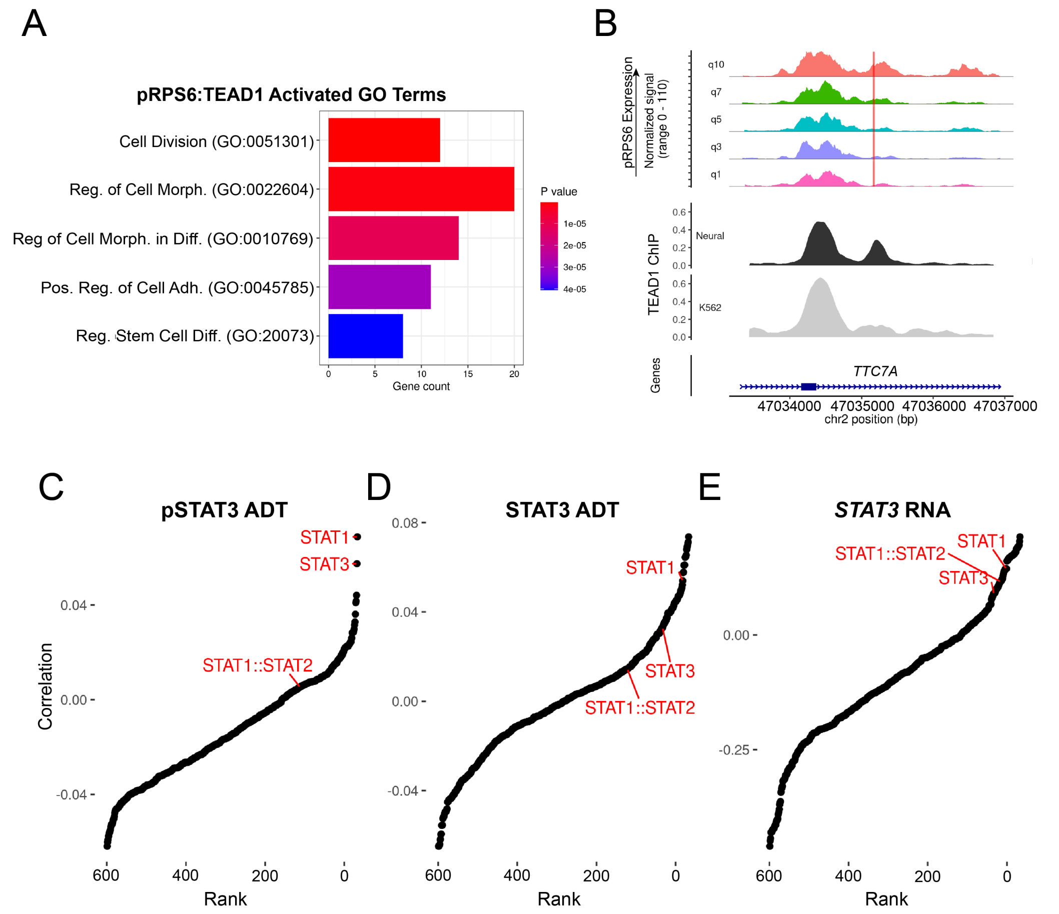

Supplementary Fig. 14: Supporting Information for Figure 6E-I. **a)** Bar plot of the top 5 most significant gene ontology categories associated with the top TEAD1 activated peak-gene links that are associated with pRPS6. **b)** Coverage plot example of cells ordered by pRPS6 expression compared to peaks from a publically available TEAD1 ChIP-seq dataset from human glioblastoma (neural) and K562 cells. **c)** Rank-correlation plot of pSTAT3 ADT signal vs. TF motif accessibility with the STAT1 and STAT3 motifs highlighted. **d)** Same as in (c) but STAT3 ADT vs. TF motif accessibility. **e)** Same as in (c) but STAT3 RNA vs. TF motif accessibility.
