# Supplementary material for: Phospho-seq: integrated, multi-modal profiling of intracellular protein dynamics in single cells
Source: Nat Commun. 2025 Feb 4;16:1346. doi: 10.1038/s41467-025-56590-7 (PMC11794950; doi:10.1038/s41467-025-56590-7)
Supplement: Supplementary file 2 — Description of Additional Supplementary Files [file 41467_2025_56590_MOESM2_ESM.pdf]

File Name: Supplementary Data 1
Description: Antibodies and associated sequencing indexes used in this study.

File Name: Supplementary Data 2
Description: Differential protein expression between pairs of cell types in Phospho-seq-ATAC and Phospho-seq-multi.

File Name: Supplementary Data 3
Description: Differential expression of GLI3-associated genes between GLI3 knockout and GLI3 wildtype organoids.

File Name: Supplementary Data 4
Description: Candidate cis-regulatory elements associated with transcription factors profiled in this study.

File Name: Supplementary Data 5
Description: Sequencing metrics for each experiment in this study.

File Name: Supplementary Data 6
Description: Gene ontology of cis-regulatory element associated genes for each transcription factor profiled.
